# Supplementary material for: Gene Expression Profiling of NFATc1-Knockdown in RAW 264.7 Cells: An Alternative Pathway for Macrophage Differentiation
Source: Cells. 2019 Feb 7;8(2):131. doi: 10.3390/cells8020131 (PMC6406727; doi:10.3390/cells8020131)
Supplement: Supplementary file 1 [file cells-08-00131-s001.pdf]

Table S1. List of genes from untransfected RAW 264.7 cells.

Gene Symbol	Fold change	Gene Symbol	Fold change	Gene Symbol	Fold change	Gene Symbol	Fold change	Gene Symbol	Fold change	Gene Symbol	Fold change
Acp5	41.89	Creb1	1.18	Gtf2b	-1.14	Ltbp2	6.05	PPARa	1.53	Tbp	1.19
Adcy10	-1.03	Crebbp	1.25	Gtf2f1	1.20	Mab21l2	1.84	PPARg	-1.53	Tcf7l2	-1.52
Alox12	-6.37	Crtap	3.49	Hand1	-1.37	Max	1.36	Prl	-1.03	Tfap2a	3.09
Alox15	-47.76	Ctnnb1	-1.33	Hand2	1.06	Mef2a	-1.60	Pth	2.00	Tgfb1	1.26
Alox5	-36.52	CtsK	-1.32	Hdac1	1.43	Mef2b	-3.15	Pth1r	2.38	Tgif1	1.55
Alpl	-1.03	Cyp17a1	-26.60	Hif1a	1.39	Mef2c	-2.98	Pthlh	-1.03	TIMP2	-2.22
Ar	-1.37	Cyp19a1	-1.03	Hnf1a	3.59	MMP2	-2.23	Rb1	-2.65	Tnfaip3	-1.64
Arnt	1.08	DKK1	-1.03	Hnf4a	-1.37	Mstn	-1.03	Rel	-1.41	Tnfrsf11a	5.26
Atf1	1.35	Dbp	1.20	Hoxa5	-1.37	Mthfr	1.39	RelA	1.29	Tnfrsf11b	-1.03
Atf2	-1.40	Dr1	1.14	Hsd11b1	19.75	Myc	108.70	Runx2	1.05	Tnfrsf1b	-4.27
Atf3	-652.60	E2f1	1.68	Hsf1	1.47	Myf5	2.58	Sfrp1	-1.03	Tnfsf11	2.42
Atf4	-1.76	E2f6	2.06	Idi1	-2.89	Myod1	1.79	Sfrp4	-1.03	Trp53	1.70
Bglap	13.96	Egr1	-144.98	Igf1	-3.43	Nanos2	1.01	Shbg	266.84	Tshr	1.77
Bmp2	-4.15	Enpp1	9.27	Igfbp2	-1.03	NFAT5	1.36	SMAD1	-1.29	Twist1	51.33
Bmp7	-1.03	Esr1	-2.78	IL15	2.50	NFATc1	14.63	SMAD4	-1.14	Vdr	-103
Calca	-1.03	Esr2	-1.03	IL6	-1.03	NFATc2	-1.15	SMAD5	3.14	VEGFa	-1.91
Calcr	1.96	Esrra	2.12	IL6ra	1.18	NFATc3	1.27	SMAD9	-1.37	Wnt10b	-1.02
Car2	-1.21	Ets1	1.32	Irf1	-1.81	NFATc4	-6.64	Sost	-1.03	Wnt3a	-1.03
Casr	1.97	Ets2	-2.53	Itga1	-1.03	Nfkb1	1.93	Sp1	-1.04	Yy1	1.37
Cd40	-1.11	Fgfr1	-4.50	Itgb3	-1.39	Nfyb	1.51	Sp3	1.17		
Cebpa	-1.30	Fgfr2	-1.03	Jun	-11.85	Nog	3.07	Sparc	-1.03		
Cebpb	-2.14	Fos	-1.75	JunB	-1.01	Nos3	-1.03	Spp1	-2.27		
Cebpg	1.02	Foxa2	-1.37	JunD	-2.72	Npy	-9.74	STAT1	-1.32		
Clasrp	1.79	Foxg1	8.82	Kcnh8	1.33	Nr3c1	1.21	STAT2	1.28		
Clcn7	1.10	GATA1	-1.37	Lep	-2.42	P2rx7	-1.86	STAT3	1.17		
Cnr2	-4.46	GATA2	2.59	Lrp1	1.10	P3h1	2.61	STAT4	5.18		
Col1a1	9.77	GATA3	2.23	Lrp5	1.50	Pax6	-1.02	STAT5a	-1.17		
Col1a2	5.00	Ghrh	1.57	Lrp6	1.72	Plod2	-1.17	STAT5b	-1.36		
Comt	1.72	Gli1	4.79	Lta	2.22	Pou2af1	1.20	STAT6a	1.84		

Table S2. List of genes from NFATc1-knockdown RAW 264.7 cells.

Gene Symbol	Fold change	Gene Symbol	Fold change	Gene Symbol	Fold change	Gene Symbol	Fold change	Gene Symbol	Fold change	Gene Symbol	Fold change
Acp5	-3.06	Creb1	-1.14	Gtf2b	1.8	Ltbp2	1.84	PPARa	1.97	Tbp	1.3
Adcy10	1.01	Crebbp	-1.01	Gtf2f1	1.04	Mab21l2	-1.68	PPARg	1.42	Tcf7l2	1.01
Alox12	1.01	Crtap	-1.14	Hand1	3.33	Max	1.18	Prl	1.01	Tfap2a	-10.74
Alox15	5.25	Ctnnb1	1.38	Hand2	1.63	Mef2a	1.26	Pth	-1.59	Tgfb1	1.07
Alox5	3.83	CtsK	-4.51	Hdac1	1.19	Mef2b	1.41	Pth1r	-1.08	Tgif1	1.4
Alpl	5.18	Cyp17a1	-1.64	Hif1a	1.08	Mef2c	-1.06	Pthlh	2.1	TIMP2	3.26
Ar	1.01	Cyp19a1	1.01	Hnf1a	-1.15	MMP2	1.49	Rb1	1.42	Tnfaip3	-1.13
Arnt	1.24	DKK1	1.01	Hnf4a	-1.01	Mstn	1.01	Rel	1.11	Tnfrsf11a	-1.08
Atf1	1.36	Dbp	1.28	Hoxa5	-7.29	Mthfr	-1.23	RelA	1.58	Tnfrsf11b	1.01
Atf2	1.33	Dr1	1.14	Hsd11b1	1.01	Myc	1.16	Runx2	2.88	Tnfrsf1b	1.07
Atf3	1.45	E2f1	1.36	Hsf1	-1.22	Myf5	2.54	Sfrp1	1.01	Tnfsf11	-2.22
Atf4	-1.29	E2f6	-1.15	Idi1	1.95	Myod1	3.21	Sfrp4	1.01	Trp53	1.01
Bglap	1.53	Egr1	-1.54	Igf1	3.72	Nanos2	-2.12	Shbg	3.4	Tshr	-1.26
Bmp2	-3.69	Enpp1	1.18	Igfbp2	1.01	NFAT5	1.12	SMAD1	1.39	Twist1	1.01
Bmp7	1.01	Esr1	1.01	IL15	1.44	NFATc1	-4.46	SMAD4	1.09	Vdr	-1.15
Calca	1,01	Esr2	1.01	IL6	1.01	NFATc2	1.34	SMAD5	1.13	VEGFa	-1.83
Calcr	-1.44	Esrra	-1.86	IL6ra	-1.42	NFATc3	1.03	SMAD9	-1.01	Wnt10b	1.01
Car2	1.92	Ets1	-1.7	Irf1	1.23	NFATc4	-1.32	Sost	1.01	Wnt3a	1.01
Casr	-2.61	Ets2	2.76	Itga1	1.01	Nfkb1	1.45	Sp1	1.22	Yy1	1.1
Cd40	1.95	Fgfr1	-12.61	Itgb3	-1.25	Nfyb	-1.01	Sp3	1.52		
Cebpa	1.23	Fgfr2	1.01	Jun	1.66	Nog	-1.54	Sparc	1.01		
Cebpb	1.11	Fos	1.15	JunB	-1.01	Nos3	1.01	Spp1	3.86		
Cebpg	1.15	Foxa2	1.64	JunD	1.17	Npy	1.09	STAT1	1.75		
Clasrp	-1.01	Foxg1	-1.36	Kcnh8	2.52	Nr3c1	1.28	STAT2	2.44		
Clcn7	-1.15	GATA1	-1.01	Lep	3.51	P2rx7	1.02	STAT3	1.27		
Cnr2	2.04	GATA2	32.4	Lrp1	1.25	P3h1	1.09	STAT4	-1.03		
Col1a1	-1.42	GATA3	1.01	Lrp5	-1.24	Pax6	-1.39	STAT5a	-1		
Col1a2	13.16	Ghrh	-1.49	Lrp6	-1.19	Plod2	-2.26	STAT5b	1.21		
Comt	1.02	Gli1	4.1	Lta	-1.4	Pou2af1	1.02	STAT6a	1.33		
